# H^+^-translocating pyrophosphatases in protozoan parasites

**DOI:** 10.1007/s00436-024-08362-3

**Published:** 2024-10-18

**Authors:** Karina Araujo-Ruiz, Ricardo Mondragón-Flores

**Affiliations:** Departamento de Bioquímica, Centro de Investigación y de Estudios Avanzados del Instituto Politécnico Nacional, Av. IPN 2508 Col. Zacatenco, Ciudad de México, 07360 México

**Keywords:** H + -PPase, Protozoan, Parasites, Pyrophosphatase, mPPase

## Abstract

Integral membrane pyrophosphatases (mPPases) hydrolyze pyrophosphate. This enzymatic mechanism is coupled with the pumping of H + and/or Na + across membranes, which can be either K + -dependent or K + -independent. Inorganic proton–translocating pyrophosphatases (H + -PPases) can transport protons across cell membranes and are reported in various organisms such as plants, bacteria, and protozoan parasites. The evolutionary implications of these enzymes are of great interest for proposing approaches related to the treatment of parasitic of phytopathogenic diseases. This work presents a literature review on pyrophosphate, pyrophosphatases, their inhibitors and emphasizes H + -PPases found in various medically significant protozoan parasites such as *Toxoplasma gondii*, the causative agent of toxoplasmosis, and *Plasmodium falciparum*, the causative agent of malaria, as well as protozoan species that primarily affect animals, such as *Eimeria maxima* and *Besnoitia besnoiti*.

## Introduction

Inorganic pyrophosphatases (PPases) are enzymes that regulate the intracellular concentration of pyrophosphate (PPi) in both prokaryotic and eukaryotic organisms. The importance of PPases involves essential reactions such as the synthesis of nucleic acids (Heinonen 2001). Since PPi is formed in large quantities as a by-product of biosynthetic reactions, PPases catalyze the hydrolysis of PPi into two molecules of Pi, thus balancing the intracellular concentration of PPi (Kajander et al. 2013; Rea and Poole 1993). PPases are classified into two general categories, soluble pyrophosphatases (sPPases) and integral membrane pyrophosphatases (mPPases), and are subclassified according to their cofactor, structure, and substrate type (Daouda et al. 2017; Gutiérrez-Luna et al. 2018; Holmes et al. 2022). The mPPases are a group of proteins that are shared between eukaryotic and prokaryotic species, which is indicative that they have undergone a long evolutionary process. H^+^-PPases belong to the mPPases that upon hydrolyzing PPi transport H^+^ and generate an electrochemical gradient across membranes in plant vacuoles (Gutiérrez-Luna et al. 2018) and membranes of acidocalcisomes in protozoan parasites (Marchesini et al. 2002; Miranda et al. 2008). H^+^-PPases are found in parasites of human interest such as *Toxoplasma gondii*, the causative agent of toxoplasmosis and *Plasmodium falciparum*, the causant of malaria as well as in species that mainly affect animals, such as *Eimeria maxima* and *Besnoitia besnoiti* (Liu et al. 2014; Luo et al. 1999; Marchesini et al. 2000; Miranda et al. 2008). Since mPPases are not found in animals or humans, H + -PPases have been considered as target enzymes in the design of therapeutical approaches against livestock and human pathogens. Here, we review the general characteristics of PPi and PPases, and how mPPases are shared between eukaryotic and prokaryotic species, and a detailed analysis on H + -PPases in protozoan parasites is presented.

### Inorganic pyrophosphate and pyrophosphatases

Inorganic pyrophosphate (PPi) is a simple chemical compound; it is composed of two inorganic phosphate (Pi) molecules containing a high-energy phosphoanhydride bond (Rea and Poole 1993). Because of the high affinity of PPi for Mg^2+^ (log Ks = 6.0), the majority of PPi is probably found forming a 1:1 complex with this metal (Canfield et al. 2005). PPi is a by-product of anabolism generated by ATP-specific substrate activation reactions such as (1) carboxylic acid activation: (adenosine triphosphate (ATP) + R-COO + coenzyme A → R-CO-coenzyme A + adenosine monophosphate (AMP) + PPi); (2) aminoacyl-tRNA synthesis: (ATP + an amino acid + tRNA → aminoacyl-tRNA + AMP + PPi); (3) coenzyme synthesis: NAD^+^  + biosynthesis (ATP + deamidoNAD^+^  + ammonia → NAD^+^  + AMP + PPi or ATP + diamidoNAD^+^  + L-glutamine → NAD^+^  + L-glutamate + AMP + PPi); (4) phosphoenolpyruvate synthesis in C4 photosynthesis: (ATP + pyruvate + orthophosphate → phosphoenolpyruvate (PEP) + AMP + PPi); (5) sulfate activation: (ATP + sulfate → adenosine phosphate sulfate (APS) + PPi); (6) de novo pyrophosphate synthesis via PPi synthesis using proton motive force: (2 orthophosphate → PPi + H_2_O); among other biosynthetic reactions (Heinonen 2001). The accumulation of PPi in large amounts is toxic to the cells, probably because is unfavorable for the reactions that generate this compound (Farquharson 2018) so, one mechanism to regulate PPi levels is the hydrolysis catalyzed by pyrophosphatases (PPases) (McIntosh and Vaidya 2002).

It has been reported that the concentration of PPi in animal cells ranges between 0.1 and 2 mM, while in the cytoplasm of plant cells it is between 0.2 and 0.3 mM (Weiner et al. 1987). PPi concentration in mitochondria ranges ± 0.2 mM, in vacuole ± 2.2 µM, and in chloroplast < 1 µM (Takeshige and Tazawa 1989). These PPi values are comparable with those reported for other intermediates of energy metabolism, such as ATP (2 to 10 mM) (Kajander et al. 2013; Rea and Poole 1993).

In various microorganisms, the glycolytic pathway includes PPi-dependent reactions. For example, in some bacteria, the main glycolytic pathway requires enzymes such as phosphofructokinase (PFK) and pyruvate phosphate dikinase (PPDK), which couple PPi hydrolysis to favor the reactions they catalyze so that, apparently, PPi-dependent glycolysis is an alternative route in fermenting bacteria in situations where ATP levels are low (Heinonen 2001). However, it has also been found in Apicomplexa parasites such as *T. gondii* and *Cryptosporidium parvum* that PFK expression employs PPi to drive fructose-6-phosphate phosphorylation (Yang et al. 2022). In addition, *T. gondii* uses PPi instead of ATP as an energy donor in at least two reactions: (a) in PFK and (b) in H^+^ translocation in acidocalcisomes using ion gradient energy (Pace et al. 2011).

**Fig. 1:**
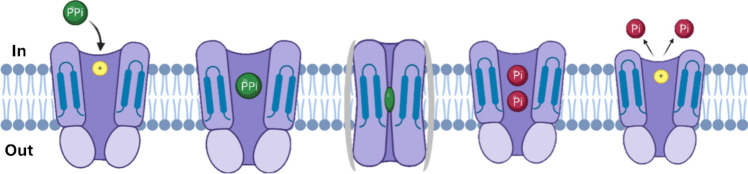
Schematic representation of the action of the membrane pump PPase on the hydrolysis of PPi. PPi is docked by charge interactions between the transmembrane passes of PPase. Its hydrolysis occurs inside the molecule, generating two Pi molecules that are released into the cytoplasm for their use in various biochemical reactions. The mPPases upon hydrolyzing PPi generate a proton motive force. The proton motive force (Δ*p*) is the sum of two components: the difference in H^+^ concentration (ΔpH) and the difference in positive charge concentration (Δ*Ψ*). Image was created with BioRender.com

As described above, most PPi comes from anabolism biosynthetic reactions. However, PPi can also be synthesized directly from two orthophosphates using ion gradient energy (Heinonen 2001). In this regard, some photosynthetic bacteria use visible light energy for proton motive force (Δ*p*, also known as proton electrochemical gradient, Δ*µ*H^+^) over the membrane in order to produce ATP or PPi through reactions catalyzed by ATPases and PPases (Fig. 1), (Remy and Gerwert 2003).

Pyrophosphatases (PPases) are the enzymes responsible for the reversible hydrolysis of the phosphoanhydride bond in PPi, yielding two molecules of inorganic phosphate (Pi). Removal of PPi allows shifting anabolic reactions towards nucleic acid and protein biosynthesis, and allowing recycling of Pi by its incorporation into ADP to form ATP (Kajander et al. 2013). PPases are classified into two categories, soluble PPases (sPPases) and membrane integral PPases (mPPases) (Kellosalo et al. 2012; Serrano et al. 2007), and are subclassified as follows.

### Soluble pyrophosphatases

Soluble pyrophosphatases (sPPases) are cytosolic proteins that are subdivided into Families I, II, and III, which are evolutionarily unrelated soluble proteins (Holmes et al. 2019). In terms of their structure, Family I harbor dimeric sPPases with a single domain, and the main cofactor for these enzymes is Mg^2+^. Family II sPPases have two domains per subunit and employ Mn^2+^ as a cofactor (Jamwal et al. 2017).

**Fig. 2:**
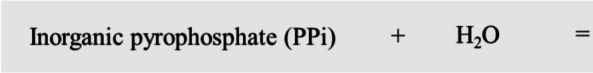
Reaction scheme of pyrophosphatases. The reversible hydrolysis of the phosphoanhydride bond (*) of PPi is shown

It has been reported that Family I and II sPPases are responsible for eliminating excess PPi waste from the cytoplasm and possess catalytic PPi hydrolysis activity of about 200 molecules per second for Family I and about 2000 molecules per second for Family II (Kajander et al. 2013). Mg^2+^-dependent sPPases hydrolyze PPi in 2 Pi; the energy of the anhydride bond is lost as heat increasing the exergonic reaction. On the other hand, the orthophosphate production reaction, due to the over stabilization of PPi by one or two Mg^2+^ cations, forms 1:1 and 1:2 complexes between both species, respectively (Primo et al. 2019). The kinetic of this reaction involves the binding of the substrate to a complex made with the Mg^2+^, the cleavage of the P-O bond (Fig. 2) by the direct effect of water molecules, and the gradual dissociation of two phosphate molecules. All these steps are easily reversible, and they allow the formation of a considerable amount of PPi bound to the enzyme. It is from this reaction that is possible to explain the rapid oxygen exchange between the ^18^oxygen isotope [^18^O] bound to Pi and [^16^O] of water in a process catalyzed by sPPases (Baykov et al. 2000). This is supported by the finding of Cohn (1958) that inorganic pyrophosphatases catalyze a rapid exchange of oxygen between inorganic orthophosphate and of water oxygen (Janson et al. 1979).

The Family III of sPPases is poorly explored and has only been reported their presence in some bacterial species, such as *Thermococcus onnurineus* NA1, where the dimer binding motifs are replaced by Trp and Gly, respectively (Lee et al. 2009). However, so far, this Family appears to be modified to haloalkane dehalogenase so, they operate by a separate mechanism to Families I and II of sPPases (Daouda et al. 2017; Lee et al. 2009).

sPPases have been identified in prokaryotes and in the cytosol of various eukaryotes, including animals, plants, parasites, and fungal cells (Jamwal et al. 2017; Kieber and Signer 1991; Sivula et al. 1999).

**Fig. 3:**
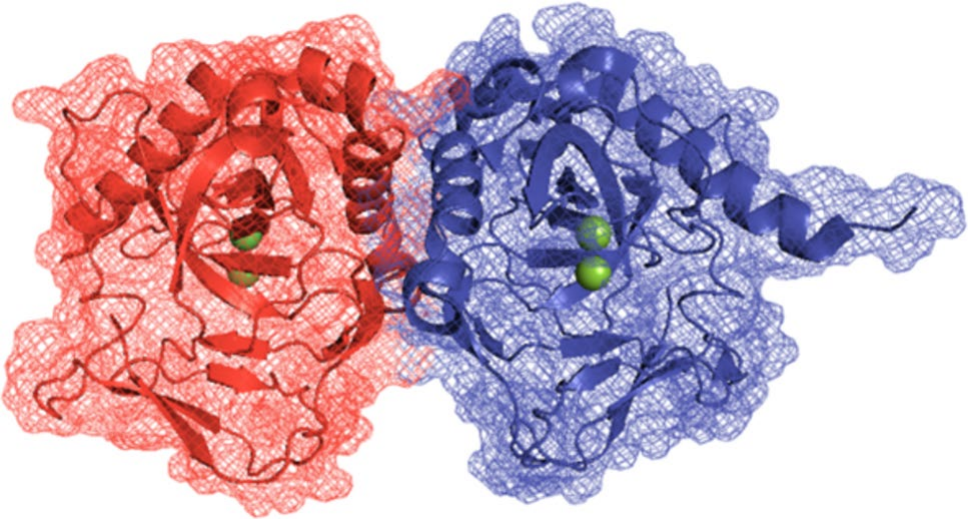
Soluble pyrophosphatase of *Toxoplasma gondii*. TgPPase. The crystallized structure has 235 amino acids and a resolution of 2.35 Å (PDB: 5WRT). Here is shown as a monomeric protein with an *αβ* structure, shown in red and blue respectively, each with two Mg^2+^ ions (green spheres) in its active site, where they are coordinated with the binding motifs (M1 and M2) of Asp190, Asp195, and Asp227. Image generated in PyMOL

In the case of protozoan parasites, *T. gondii* has a sPPase known as TgPPase, which is an enzyme that contains two Mg^2+^ ions in its active site, coordinated by two binding motifs (M1 and M2) through the amino acids Asp190, Asp195, and Asp227 (Jamwal et al. 2017). The structure of the TgPPase (Fig. 3) has been crystallized and has a resolution of 2.35 Å, a molecular weight (MW) of 53.63 kDa, and is available in the Protein Data Bank (PDB) with ID: 5WRT (Jamwal et al. 2017). In addition, TgPPase has been reported to regulate the cytosolic level of PPi in the parasite (Pace et al. 2011).

### Integral membrane pyrophosphatases

**Fig. 4:**
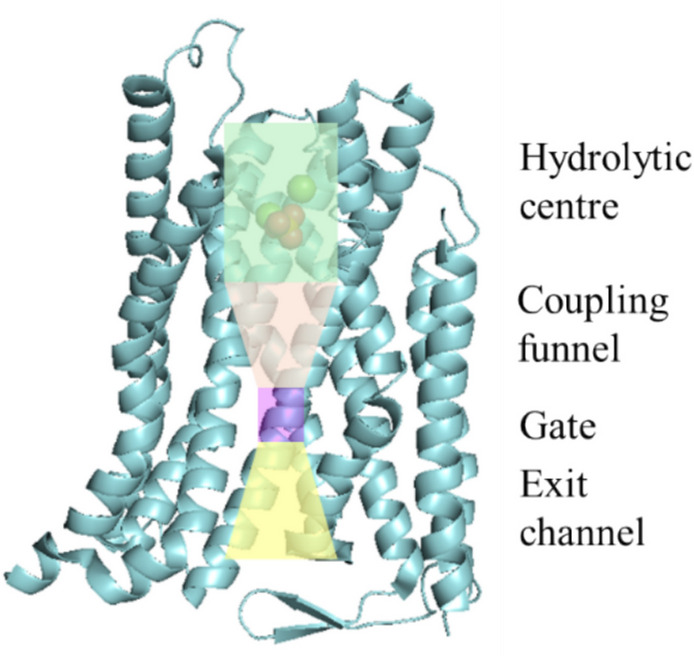
Cross-sectional view of the composition of the transmembrane passages of H^+^-PPase from *Vigna radiata*. The different substructures of the active site are shown: the hydrolytic center, the coupling funnel, the ionic gate, and the exit channel. Helices 4, 7, 9, 10, and 14 have been removed for clarity. Image created in PyMOL

The mPPases are homodimeric proteins and are the only Family of membrane-embedded PPases with transmembrane helices (TMHs), between 15 and 17 per subunit, and a MW between 70 and 81 kDa (Holmes et al. 2019; Kellosalo et al. 2012). A single subunit is composed by two concentric rings of TMHs. Four catalytic regions are constituted inside the ring: (1) the hydrolytic center; (2) the coupling funnel; (3) the ionic gate; and (4) the exit channel (Fig. 4) (Holmes et al. 2022; Li et al. 2016). Because these proteins are embedded in or associated with membranes, they function as primary ion pumps that couple PPi hydrolysis to the movement of H^+^ (H^+^-PPase) and/or Na^+^ (Na^+^-PPase), and thus generate a membrane potential that contributes to a number of cellular functions such as the regulation and energization of organelles such as the acidocalcisomes in parasites and the vacuole in the case of plants (Kajander et al. 2013; Luoto et al. 2013).

The catalytic activity of mPPases differs by orders of magnitude concerning sPPases as mPPases are slower at hydrolyzing about 10 PPi molecules per second compared to sPPases that hydrolyze around 2000 molecules per second (Kajander et al. 2013). Currently, mPPases have been classified according to their function into seven different groups: in terms of catalytic activity (1) K^+^-dependent and (2) K^+^-independent. Based on pumping specificity is shown as follows: (3) H^+^ pumping only; (4) Na^+^ pumping only; (5) dual H^+^/Na^+^ pumping; (6) dual H^+^/H^+^ pumping; and finally (7) Na^+^-regulated (Holmes et al. 2022; Strauss et al. 2018).

### Integral membrane pyrophosphatases are shared between species

One of the principles of molecular biology indicates that all living organisms have evolved from a single common ancestral cell that lived more than three billion years ago (Weiss et al. 2018). Because it gave rise to all known living organisms, this ancient cell is often referred to as the “last universal common ancestor (LUCA)” (Doolittle 1999; Penny and Poole 1999). Based on the above, it is not trivial to consider that some proteins, such as mPPases, have been conserved between species, with some structural or functional modifications. Since the discovery in 1967 of the H^+^-transporting mPPase (H^+^-PPase) in the photosynthetic purple bacterium *Rhodospirillum rubrum*, PPi was evidenced as the main product of photophosphorylation in chromatophores and initiated the study of ion gradients and ∆*p* in this bacterium (Baltscheffsky 1967).

Later studies revealed that plants also possessed H^+^-PPases in their vacuolar membrane, but K^+^-dependent (Karlsson 1975). This was a watershed in the differentiation of the mPPases, since it was discovered that plant H^+^-PPases needed K^+^ ions to function, whereas the H^+^-PPases of *R. rubrum* bacteria did not and consequently, they were categorized into two phylogenetically distinct types of enzymes: K^+^-dependent and K^+^-independent.

Additionally, a mPPase was detected for the first time in the acidocalciosomes of the protist *Trypanosoma cruzi* (Scott et al 1998).

Through the development of sequencing and cloning techniques, it was possible not only to identify the mPPase genes but also to express the proteins in organisms such as *Escherichia coli* and *Saccharomyces cerevisiae*. Based on these constructs, the functional divergence of mPPases was understood. In this context, Belogurov and Lahti (2002) achieved Na^+^ activation of the mPPase in the thermophilic bacterium *Carboxydothermus hydrogenoformans*, as well as PPi hydrolysis and H^+^ translocation by the recombinant enzyme inserted into *E. coli* membrane vesicles. Through phylogenetic analyses, they identified that K^+^-independent H^+^-PPases possessed conserved Lys and Thr residues, absent in K^+^-dependent H^+^-PPases (Belogurov & Lahti 2002). K^+^-dependent mPPases contain Ala in the position occupied by Lys in K^+^-independent H^+^-PPases; the positively charged amino group of Lys replaces K^+^ in these latter mPPases (Li et al. 2016).

Subsequent studies showed that mPPases not only transported H^+^, but were also capable of transporting Na^+^, opening up a field of research for Na^+^-PPases. Additionally, this type of transport was demonstrated for the first time in bacteria such as *Moorella thermoacetica*, *Thermotoga maritima*, and *Methanosarcina mazei* (Malinen et al. 2007). Subsequent works established Na^+^-PPases as a widespread subfamily independent of H^+^-PPases (Luoto et al. 2011). The mPPases that transport Na^+^ ions are characterized as belonging to the K^+^-dependent subfamily, whereas mPPases that pump H^+^ can be found in both families (Malinen et al. 2007).

The study of mPPases has provided insight into the evolution and modification of these proteins functionally as ion pumping proteins (Luoto et al. 2011, 2013). In the bacterium *Chlorobium limicola*, mPPases are capable of simultaneously transporting both H^+^ and Na^+^ ions, which, presents pumping through two Na^+^-binding sites in the channel: one associated with the gate that controls enzyme activities and another that controls only activity of H^+^ ions transport (Luoto et al. 2011).

The hydrolysis of PPi through mPPases can be coupled to the translocation of H^+^ and/or Na^+^ across membranes, since mPPases are functionally analogous to F-type ATPases, additionally they catalyze the direct attack of water molecules on a phosphate atom, without forming a phosphorylated intermediate (Holmes et al. 2019). The high-energy phosphoanhydride bonds of ATP have been used as the main energy exchange currency among biochemical reactions (Drozdowicz and Rea 2001; Rea and Poole 1993). However, the discovery of abundant polyphosphate reserves and pyrophosphate-hydrolyzing enzymes (PPases) in several organisms has proposed that the high-energy phosphoanhydride bonds of PPi or polyphosphate (polyP) may serve as important alternative or complementary energy sources under stress conditions, or in organisms where metabolic functions must be tightly regulated (Folgueira et al. 2021; Holmes et al. 2019).

The presence of mPPases, in both parasites and plants, indicates that these proteins have been conserved among eukaryotic species, since they are found in the vacuolar membranes (tonoplast) of higher plants, algae, and protozoa, as well as in bacteria and archaea (Baltscheffsky 1967; Baltscheffsky et al. 1966; Holmes et al. 2019; McIntosh et al. 2001).

Based on the discovery of H^+^-PPases in different organisms, Baltscheffsky et al. (1999) performed a sequence analysis among algae and plant species possessing H^+^-PPases such as *Acetabularia acetabulum*, *Chara corallina* (Takeshige and Tazawa 1989), *Oryza sativa*, *Beta vulgaris* (Karlsson 1975), *Nicotiana tabacum*, *Cucurbita moschata*, *Vigna radiata* (Li et al. 2016), *Hordeum vulgare*, and *Arabidopsis thaliana* (Mitsuda et al. 2001; Segami et al. 2018) and performed consensus sequences with respect to bacterial H^+^-PPases and *R. rubrum* and *T. maritima*. Sequence analysis of the H^+^-ATPases, revealing that hydrolysis of PPi to provide energy, may have occurred prior to the adoption of ATP as a universal energy currency. Thus, that work suggested that mPPases may have been the first enzyme to couple phosphoanhydride bond hydrolysis to the chemiosmotic potential (Baltscheffsky et al. 1999).

In this sense, the work by Drozdowicz and Rea (2001) expanded the sequence analysis of H^+^-PPases to include plants, algae, bacteria, and protozoan parasites. The analysis showed that the H^+^-PPases of the analyzed parasites such as *T. gondii*, *P. falciparum*, *Trypanosoma brucei*, and *T. cruzi* have a greater identity with respect to the H^+^-PPases of the plants.

**Fig. 5:**
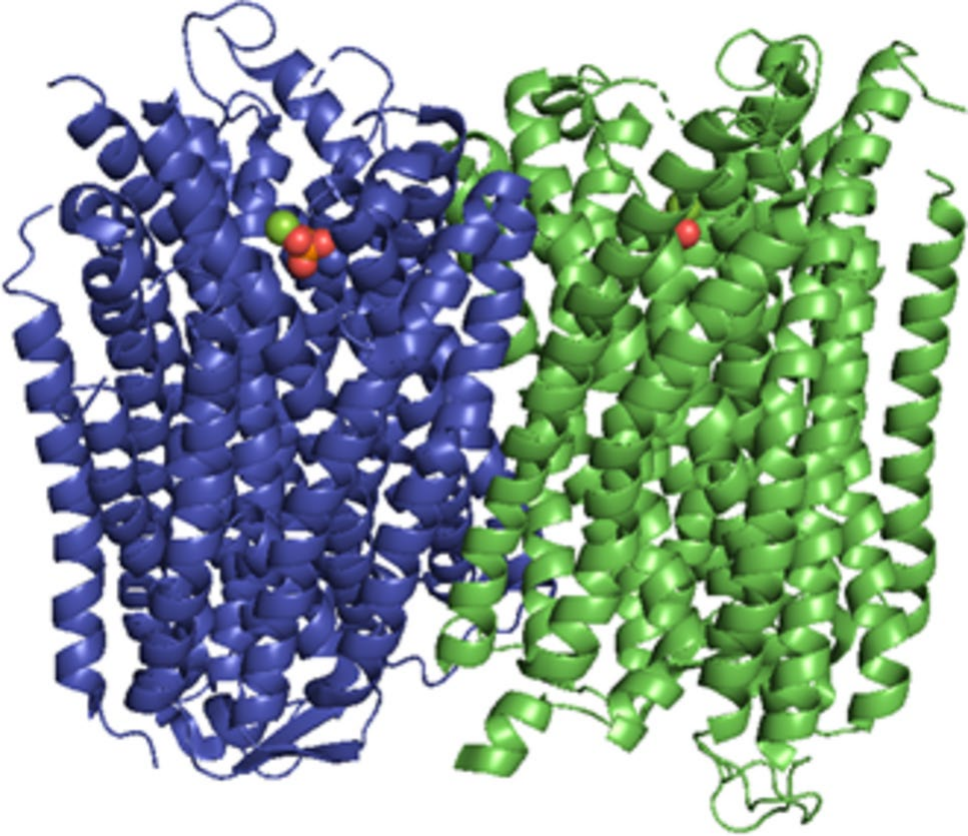
H^+^-PPase from *Vigna radiata* (PDB: 5GPJ). The dimeric structure is depicted in blue and green, each with 13 transmembrane helices (TMH). The structure of the ligand bound to the active site is also shown between the TMH oxygen (O_2_) atoms in red; phosphorus (P) in orange; Mg^2+^ ion in green. Image created in PyMOL

In higher plants, H^+^-PPases have been reported to have two main physiological functions: the hydrolysis of PPi in the cytosol and the acidification of vacuoles (Segami et al. 2018), where PPi is hydrolyzed by vacuolar H^+^-PPases (V-PPases) that conserve energy and take advantage of the free energy of PPi hydrolysis to establish transmembrane H^+^ gradients (Au et al. 2006). In the specific case of the bean *V. radiata*, it possesses an H^+^-PPase identified as VrPPase (Fig. 5) that has been crystallographically characterized with a resolution of 3.50 Å (PDB: 5GPJ) (Li et al. 2016). The mPPase of *V. radiata* has been used as a template for topological predictions of the proton-translocating pyrophosphatase of *P. falciparum* (McIntosh and Vaidya 2002).

In addition, the existence of H^+^-PPases has been demonstrated in vacuoles (tonoplasts), Golgi apparatus, plasma membrane, mitochondria, and chloroplasts; although, the corresponding genes have not always been identified (Mitsuda et al. 2001; Ratajczak et al. 1999; Segami et al. 2018; Zancani et al. 1995). On the other hand, in parasites, the function of proton-pumping pyrophosphatases (H + -PPases) has been associated with establishing H + gradients across the plasma membrane in energy-limiting conditions. In such conditions, parasites undergo morphological and molecular changes, for example, during stages of interconversion that occur during their life cycle or in the encyst process (Folgueira et al. 2021).

### H^+^-PPases in protozoa

The mPPases have been reported in different members of the eukaryotic evolutionary lineages, ranging from unicellular algae to higher plants such as *A. thaliana* that presents an mPPase in the Golgi apparatus (Mitsuda et al. 2001). They are also found in acidocalcisomes of protist such as *T. cruzi* (Moreno and Docampo 2009), *T. gondii* (Rodrigues et al. 2000), and *Plasmodium berghei* (Marchesini et al. 2000). In *Bacteroides vulgatus*, they have been reported in the inner cell membrane (Luoto et al. 2013).

Different human and animal infectious diseases of veterinary importance are caused by protozoan parasite, e.g., malaria is caused by the parasite *Plasmodium* spp., toxoplasmosis by *T. gondii*, trypanosomiasis by *Trypanosoma* spp., and leishmaniasis by *Leishmania* spp. Each of these diseases poses a high prevalence and risk of mortality or has been associated with other diseases (Holmes et al. 2019; Shah et al. 2016). The life cycle of protozoan parasites often involves transitions between vectors and hosts, as well as between intracellular and extracellular environments, meaning that they must survive and adapt to different conditions (Blader et al. 2015). In terms of the function of mPPases, the most relevant change to overcome is the difference in osmotic pressure that cells experience in these different environments (Holmes et al. 2019). The main mechanism of protozoa to regulate internal osmotic pressure involves acidocalcisomes (Docampo and Moreno 2011). The translocation of H + across membranes is a topic of interest in the study of parasites since the location of H + -PPases has helped in the study of the effects of different antiparasitic drugs (Folgueira et al. 2021; Valueva et al. 2020). In this sense, studies-based knock-down and knock-out in parasites such as *T. brucei* (Lemercier et al. 2002), *T. gondii* (Liu et al. 2014), and *P. falciparum* (Zhang et al. 2018) have demonstrated that mPPases are required to maintain acidocalcisomes acidification, parasite virulence, and in vitro growth of the asexual stages of these parasites.

*Philasterides dicentrarchi* is a parasite of aquaculture importance, because it affects economically important fish such as snook. *P. dicentrarchi* is a microaerophilic, euryhaline organism, capable of surviving in hypoxia and hypo-salinity conditions (Mallo et al. 2013). This protozoan has been reported to express two isoforms of H + -PPases, classified as VP1 and VP2, which were generated by alternative splicing (Mallo et al. 2015). Regarding their subcellular distribution, H^+^-PPases are present in the membranes of phagocytic vacuoles and the alveolar sacs of *P. dicentrarchi* (Folgueira et al. 2021). Alternative splicing has also been reported as the mechanism responsible in plants for the generation of two isoforms of H^+^-PPases (Maeshima 2000).

Furthermore, *T. cruzi* possesses an H + -PPase in the Golgi apparatus and in vesicles originating from the plasma membrane (Martinez et al. 2002). In addition, in a previous study, the presence of an H^+^-transport pump in the acidocalcisomes of this parasite was reported (Scott et al. 1998). In *T. brucei*, which uses an insect as a vector for its transmission in humans and animals, an H^+^-PPase has been reported in the acidocalcisomes of the parasite (Lemercier et al. 2002). In these two species of *Trypanosoma*, no modifications or isoforms by splicing of H^+^-PPases have been identified.

As part to the results obtained from genome resolution and data mining, it has been possible to know the sequence of the mPPases. Through the comparative genomic analysis of three *Leishmania* species (*L. infantum*, *L. braziliensis*, and *L. major*) (Peacock et al. 2007), it was reported that *L. infantum*, the only *Leishmania* species that generates the disease known as canine leishmaniasis and that also affects humans, has an H^+^-PPase; although, its subcellular location has not yet been described. The same case presents the ciliate parasite *Stentor coeruleus* which presents a trumpet morphology; to date, its genome has been elucidated, and as part of the analysis of the sequences it could be demonstrated that this parasite also possesses an H^+^ translocating mPPase, although with unknown subcellular location (Slabodnick et al. 2017).

The parasite *B. besnoiti* is a protozoan belonging to the phylum Apicomplexa where the parasites *T. gondii*, *Cystoisospora suis*, and *Neospora caninum* are also classified. *B. besnoiti* is of great veterinary medical importance, since it mainly affects cattle; although, it can also affect humans (Velásquez et al. 2020). The genome of this parasite is resolved and genes coding for an H^+^-PPase have been identified among the annotations (Schares et al. 2017); although the subcellular location in the parasite is not reported, the sequence of the enzyme is available in the UniProt database: A0A2A9MPV3. A similar case presents the Apicomplexa parasite *C. suis*, which causes the porcine cystoisosporosis disease, that presents different stages in its life cycle. Additionally, the cloning, expression, and sequencing of various proteins of this organism has been carried out, and its genome has also been reported, as well as the sequence of the H + PPases through UniProt: with the code A0A2C6KPM9 (Schares et al. 2017).

*Toxoplasma* is considered as one of the model organisms of Apicomplexa parasites, and has generated extensive research on PPases, including the report of the crystallized structure of an sPPase, named as TgPPase (PDB:5WRT), which is present in the cytoplasm of the parasite regulating cytosolic levels of PPi (Pace et al. 2011). Two proton pumps, the V-H + -ATPase and the vacuolar H + -PPase (TgVP1), have been reported in *T. gondii* acidocalcisomes that contribute to their acidification, allowing pH regulation, osmotic homeostasis, and Ca^2+^ signaling (Miranda et al. 2008; Rodrigues et al. 2000). Furthermore, the presence of an H + -PPase identified as TgVP1 has been reported in a novel lysosome-like compartment called plant-like vacuole (PLV) or vacuolar compartment (VAC) (Liu et al. 2014). On the other hand, the parasite *N. caninum* possesses an H^+^-PPase that is inserted into the parasite membrane through its respective transmembrane passages (Reid et al. 2012). Another Apicomplexa protozoa of medical relevance in humans is *Cyclospora cayetanensis*, whose life cycle is similar to *T. gondii* in that it forms tissue cyst to safeguard the parasites inside (Ceridwen et al. 2021). *C. cayetanensis* possesses a genome with 144 tRNA genes encoding 7457 proteins, including an H^+^-PPase reported in the parasite cell membrane (Liu et al. 2016). The resolved genome of *C. cayetanensis* has allowed the development of comparative research based on genomics between components of the metabolism and components related to the invasion process, including the characterization of surface antigens involved in the recognition and adhesion to the host cell by molecules such as the SRS proteins (SAG1-related sequences) of *T. gondii* and the T4A-like surface antigens of *Eimeria tenella* (Reid et al. 2012). Liu et al. (2016) reported that *C. cayetanensis* does not possess any gene cluster encoding T4A-like surface antigens previously identified in *E. tenella* (Reid et al. 2014); although, SRS proteins do participate in the invasion process as in the case of *T. gondii* and *E. tenella* (Liu et al. 2016).

Malaria, caused by *Plasmodium* spp. parasite, is a serious infectious disease in humans and of global importance; however, different species of *Plasmodium* spp., such as *Plasmodium yoelii* and *P. berghei* that infect rodents, have been used to study the cellular and molecular characteristics of the host-parasite relationship. (Pichugin and Krzych 2015; Yuguchi et al. 2021). In humans, *P. falciparum* is the main variant associated with malaria (Béré et al. 2019); although, there are other species that also infect humans such as *P. ovale*, *P. gonderi*, *P. knowlesi*, and *P. relictum* (Garrido-Cardenas et al. 2019; Milner 2018). All these *Plasmodium* variants have a reported H^*^-PPase; although in not all cases, its cellular locations are known. In the case of *P. falciparum*, mPPases have been shown in the plasma membrane of the parasite, identifying two types of H^+^-PPases, K^+^-dependent and K^+^-independent, named PfVP1 and PfVP2, respectively (McIntosh et al. 2001). Although an H^+^-PPase has also been reported in the membrane of *P. falciparum* acidocalcisomes (Riel et al. 2002). This cellular location coincides with that reported for *T. gondii* (Miranda et al. 2008).

In another group of parasites related to the Phylum Apicomplexa, *Vitrella brassicaformis* is found, which possesses an H^+^-PPase, reported in the plasma membrane (Templeton and Pain 2015). Other equally Apicomplexa parasites are *Eimeria brunetti* and *E. maxima* which mainly affect poultry, causing the intestinal disease known as hemorrhagic coccodiosis (Burrell et al. 2020). According to cellular distribution predictions, the reported location for the H^+^-PPases of these parasites is in the plasma membrane (Reid et al. 2012).

The parasite *Perkinsus chesapeaki* infects mainly mollusks and is the causative agent of the disease Perkinsosis, in this case, although expression profiling by mass spectrometry has been performed and an H^+^-translocating mPPase has been reported; however, its subcellular location in the parasite has not been elucidated (Bogema et al. 2021; Fernández-Boo et al. 2014). These protozoan parasites are of great medical and veterinary relevance and all of them possess an H^+^-PPase in different subcellular locations. However, their distribution has not been demonstrated in all cases by experimental approaches with targeted antibodies or by biochemical or molecular techniques, so the presence and distribution of these mPPases is not fully known and they have only been identified by genome resolution.

Thanks to an evolutionary prediction research, it is now known that mPPases are not exclusive to parasites, but are also present in plants, algae, and bacteria. In the case of plants and protozoa, it has been shown that ion translocation by H^+^-PPases occurs under stress conditions due to lack of nutrients or morphological changes in the case of parasites (Goodenough et al. 2019).

### Pyrophosphatase inhibitors

The sPPases are inhibited with micromolar (µM) concentrations of fluorine (F^−^), so it has been employed as a monitor of PPi (Baykov et al. 1992). This ion has been tested in *S. cerevisiae* sPPase (Y-PPase) and *E. coli* PPase (E-PPase), observing an instantaneous decrease (mean time < 1 s) in the activity after addition of fluorine to the enzyme (Baykov et al. 1992). F^−^ ion has also been studied as an enzyme poison, inducing oxidative stress, hormonal disorder, and neurotoxicity in humans (Strunecka and Strunecky 2020). Sodium fluoride (NaF) is the commonly used reagent in sPPase inhibition and has been shown to inhibit firefly luciferase activity by 80% using 10 mM NaF to inhibit its sPPase (Eriksson et al. 2001). In the case of *T. gondii*, 2 mM NaF has been shown to inhibit PPi hydrolysis in tachyzoite homogenates (Rodrigues et al. 2000).

The ability of NaF to inhibit sPPases is due to the ability of the F^−^ ion to occupy the side of the water molecule, activated to perform nucleophilic attack of phosphorus, in a hydrolysis reaction that resembles a nucleophilic substitution 2 (SN2) reaction (Wu et al. 2021).

Moreover, in an early report, Eubank and Reeves (1982) found that bisphosphonates, hydrolytically stable analogues of PPi, had activity against *Entamoeba histolytica* and proposed that these compounds inhibited PPi-dependent PFK of the parasite. Subsequently, further results on *E. histolytica* were reported using various nitrogen-containing bisphosphonates. It is now known that bisphosphonates (Fig. 6) are potent inhibitors of mPPase enzymes (Ghosh et al. 2004; Park et al. 2021). The mPPases exhibit high sensitivity to the competitive bisphosphonate inhibitor 1,1-diphosphonate, aminomethylenediphosphonate (AMDP) (Baykov et al. 1993). AMDP has one methylene carbon substituted with two oxidized phosphorus atoms. The resulting structure, P–C-P, has two phosphate groups liked by non-hydrolyzable phosphoether bonds. The central carbon atom can also harbor two additional substituents via covalent bonds with other elements, such as carbon, oxygen, nitrogen, sulfur, and a halogen, giving rise to a huge variety of compounds, which can vary greatly in reactivity and chemistry (Ghosh et al. 2004; Ling et al. 2005). Different bisphosphonates, such as pamidronate, alendronate, tiludronate, risedronate, etidronate, clodronate, and ibandronate, have been studied and established as effective therapies in the treatment of bone resorption disorder, osteoporosis, and Paget’s disease, as well as myeloma and bone metastases (Russell et al. 1999). Phosphonates and bisphosphonates are chemical analogs with stability to phosphates and pyrophosphates. Bisphosphonates are especially useful as inhibitors of enzymes involved in isoprenoid biosynthesis pathways, inhibiting proton transport and PPi hydrolysis (Park et al. 2021). These drugs have also been evaluated as potent antiparasitic agents (Eubank and Reeves 1982; Ghosh et al. 2004).

**Fig. 6:**

Structure of the bisphosphonate. Two side chains R1 and R2 are shown which modify their functional properties in the different derivatives

In addition to the finding that mPPases are sensitive to inhibition by AMDP in both plant cells and photosynthetic bacterial cells (Drozdowicz et al. 2003), several studies have reported that this compound inhibits the growth of parasites such as *P. falciparum* (McIntosh et al. 2001), *T. gondii* (Rodrigues et al. 2000), and *T. cruzi* (Urbina et al. 1999). The potential for developing pharmacological derivatives of this compound or alternatives agents specifically designed to inhibit mPPases is of great interest in the search for therapeutic alternatives against intracellular parasites.

In the case of *T. gondii*, the activity of 60 types of bisphosphonates has been studied as inhibitors of the parasite’s growth in vitro to identify potential antiparasitic compounds (Ling et al. 2005). The C10 bisphosphonate has a long carbon chain and has been reported as an inhibitor of acid sphingomyelinase and as a treatment option for inflammatory lung diseases, cyst fibrosis, and atherosclerosis (Roth et al. 2009). This compound interacts with divalent metal ions and forms bidentate complexes. Bisphosphonates are inhibitors of cytosolic farnesyl diphosphate (FDP), synthase (geranyl diphosphate (GDP) and geranylgeranyl diphosphate (GGDP) synthase), and the isoprene pathway. Additionally, they have antimicrobial functionality and have been reported as inhibitors of the growth of *E. histolytica* and *P. falciparum* (Eubank and Reeves 1982; Ghosh et al. 2004).

## Conclusion

Inorganic pyrophosphate (PPi) is a by-product of various metabolic pathways, and it has been shown to be constantly regulated within cells to prevent against its toxicity. Pyrophosphatases are not merely enzymes that degrade PPi; they are also implicated in the functioning and balance of various biochemical reactions. Since their discovery, numerous studies have demonstrated the importance of these enzymes in cellular metabolism. These proteins are involved in various eukaryotic and prokaryotic organisms, which suggests, based on the published studies, that these proteins have been in constant evolution as they have been reported in bacteria, higher plants, and protozoa. Specifically, in protozoa, mPPases play an important role as energy reservoirs in acidocalcisomes, where the proton motive force generated by H^+^-PPases also supports the energization conditions in this organelle. In membranes, H^+^-PPases not only support acidification and changes in the ∆*G*°′, but thanks to evolutionary predictions tools, we now know that this feature is not exclusive to parasites. Plants also possess vacuolar and/or transmembrane mPPases, and H^+^ translocation is also activated under stress conditions, likely a result of cellular evolution stemming from the endosymbiotic theory.

Among the evolutionary implications of these enzymes is the possibility of generating new approaches for the treatment of diseases generated by protozoan parasites, since these enzymes, being embedded in membranes, could be potential targets in the search for new pharmacological alternatives for the treatment of diseases of human and veterinary interest.

## Data Availability

No datasets were generated or analysed during the current study.
